# Milk From Women Diagnosed With COVID-19 Does Not Contain SARS-CoV-2 RNA but Has Persistent Levels of SARS-CoV-2-Specific IgA Antibodies

**DOI:** 10.3389/fimmu.2021.801797

**Published:** 2021-12-23

**Authors:** Ryan M. Pace, Janet E. Williams, Kirsi M. Järvinen, Courtney L. Meehan, Melanie A. Martin, Sylvia H. Ley, Celestina Barbosa-Leiker, Aline Andres, Laxmi Yeruva, Mandy B. Belfort, Beatrice Caffé, Alexandra D. Navarrete, Kimberly A. Lackey, Christina D. W. Pace, Alexandra C. Gogel, Bethaney D. Fehrenkamp, Miranda Klein, Bridget E. Young, Casey Rosen-Carole, Nichole Diaz, Stephanie L. Gaw, Valerie Flaherman, Mark A. McGuire, Michelle K. McGuire, Antti E. Seppo

**Affiliations:** ^1^ Margaret Ritchie School of Family and Consumer Sciences, University of Idaho, Moscow, ID, United States; ^2^ Department of Animal, Veterinary and Food Sciences, University of Idaho, Moscow, ID, United States; ^3^ Department of Pediatrics, Division of Allergy and Immunology, University of Rochester School of Medicine and Dentistry, Rochester, NY, United States; ^4^ Department of Anthropology, Washington State University, Pullman, WA, United States; ^5^ Department of Anthropology, University of Washington, Seattle, WA, United States; ^6^ School of Public Health and Tropical Medicine, Tulane University, New Orleans, LA, United States; ^7^ College of Nursing, Washington State University Health Sciences Spokane, Spokane, WA, United States; ^8^ Department of Pediatrics, University of Arkansas for Medical Sciences, Little Rock, AR, United States; ^9^ Arkansas Children’s Nutrition Center, Little Rock, AR, United States; ^10^ Department of Pediatric Newborn Medicine, Brigham and Women’s Hospital and Harvard Medical School, Boston, MA, United States; ^11^ Elson S. Floyd College of Medicine, Washington State University, Spokane, WA, United States; ^12^ Department of Obstetrics, Gynecology & Reproductive Sciences, Division of Maternal-Fetal Medicine, University of California, San Francisco, San Francisco, CA, United States; ^13^ Department of Pediatrics, University of California, San Francisco, San Francisco, CA, United States

**Keywords:** antibodies, breastfeeding, COVID-19, human milk, immunoglobulins, IgA, passive immunity, SARS-CoV-2

## Abstract

**Background:**

Limited data are available regarding the balance of risks and benefits from human milk and/or breastfeeding during and following maternal infection with severe acute respiratory syndrome coronavirus 2 (SARS-CoV-2).

**Objective:**

To investigate whether SARS-CoV-2 can be detected in milk and on the breast after maternal coronavirus disease 2019 (COVID-19) diagnosis; and characterize concentrations of milk immunoglobulin (Ig) A specific to the SARS-CoV-2 spike glycoprotein receptor binding domain (RBD) during the 2 months after onset of symptoms or positive diagnostic test.

**Methods:**

Using a longitudinal study design, we collected milk and breast skin swabs one to seven times from 64 lactating women with COVID-19 over a 2-month period, beginning as early as the week of diagnosis. Milk and breast swabs were analyzed for SARS-CoV-2 RNA, and milk was tested for anti-RBD IgA.

**Results:**

SARS-CoV-2 was not detected in any milk sample or on 71% of breast swabs. Twenty-seven out of 29 (93%) breast swabs collected after breast washing tested negative for SARS-CoV-2. Detection of SARS-CoV-2 on the breast was associated with maternal coughing and other household COVID-19. Most (75%; 95% CI, 70-79%; n=316) milk samples contained anti-RBD IgA, and concentrations increased (*P*=.02) during the first two weeks following onset of COVID-19 symptoms or positive test. Milk-borne anti-RBD IgA persisted for at least two months in 77% of women.

**Conclusion:**

Milk produced by women with COVID-19 does not contain SARS-CoV-2 and is likely a lasting source of passive immunity *via* anti-RBD IgA. These results support recommendations encouraging lactating women to continue breastfeeding during and after COVID-19 illness.

## Introduction

Most studies examining milk produced by women with coronavirus disease 2019 (COVID-19) have demonstrated that it is an unlikely source of severe acute respiratory syndrome coronavirus 2 (SARS-CoV-2) maternal-to-child transmission (1–4). Nonetheless, SARS-CoV-2 has been detected in a fraction of milk samples in some studies (5–7). The reasons for these disparate findings are unknown, but it is likely that sample collection and/or analysis methodology might play a role. Additionally, several studies have shown that milk antibody titers correlate with the milk’s ability to neutralize SARS-CoV-2 infectivity (3, 8–11), thus likely offering immunological protection to the infant. Together, these findings support epidemiological evidence that breastfeeding while using appropriate hand and respiratory hygiene does not increase risk of infant SARS-CoV-2 infection (12, 13).

Individuals infected with SARS-CoV-2 typically develop a robust serum antibody response against the spike (S) glycoprotein within 2 weeks of illness onset. While early studies demonstrated that circulating levels waned by 2 months (14, 15), more recent research suggests more moderate declines with continued seropositivity at 6 to 8 months (16). Similarly, milk produced by women with COVID-19 contains substantial immunoglobulin A (IgA) targeting the S glycoprotein receptor binding domain (RBD) in the first month following infection (3, 9). However, little is known about the persistence of milk anti-SARS-CoV-2 IgA following maternal infection.

The presence of virus and anti-viral antibodies in milk contribute to the balance of risks and benefits that breastfeeding provides to infants of mothers with SARS-CoV-2 infection. The primary objective of this study was to use validated analytical methods and optimized longitudinal sampling to analyze milk produced after maternal COVID-19 diagnosis for the presence of SARS-CoV-2, as well as levels and duration of milk-borne anti-RBD IgA for up to 2 months after diagnosis. To further understand whether breast skin could be a possible source of viral RNA contamination in milk and/or represent a potential route of exposure to the infant, we also assessed the prevalence of SARS-CoV-2 on breast skin swabs collected before and after cleaning the breast.

## Material and Methods

### Experimental Design and Clinical Data/Sample Collection

This multicenter study was carried out from April to December 2020 using a repeated-measures, longitudinal design. Maternal-infant dyads were recruited through participating institutions (University of Idaho; Washington State University; University of Rochester Medical Center; University of California, San Francisco; Brigham and Women’s Hospital; University of Arkansas for Medical Sciences; Tulane University), and national social media advertising. To participate, women needed to be ≥18 years of age, lactating, have an infant less than 24 months old, and diagnosed with or tested for COVID-19 in the last seven days. No SARS-CoV-2 vaccine was available during the study period. Milk, breast skin swabs, and telephone surveys were ideally collected on three separate days during the first week post-diagnosis and again at 2-, 3-, 4-, and 8-weeks post-diagnosis. Participants self-collected milk and breast swab samples using provided collection kits, which were assembled aseptically by study personnel wearing masks and gloves. Mothers were instructed in clean techniques to obtain samples, including use of gloves and masks. Surveys included questions about COVID-19 testing results for all household members and maternal and infant COVID-19 symptoms. This multi-institutional study was reviewed and approved by the institutional review boards of the University of Idaho (20–056, 20–060), University of Rochester Medical Center (1507), University of California, San Francisco (20–30410), Brigham and Women’s Hospital (2020P000804), University of Arkansas for Medical Sciences (260939), and Tulane University (2020–602). All participants gave written informed consent.

We previously reported (3) on 37 milk samples collected from 18 women in the first week post-diagnosis; of these women, 11 were recruited to provide additional longitudinal samples up to 2 months post-diagnosis for this study. In addition, we recruited 63 additional participants for this study (**Supplementary Figure 1**). No sample size calculation was performed due to logistical considerations and lack of preliminary data. Due to the nature of conducting this research during a pandemic and because test results were often not available soon after testing, some women were recruited after being tested but prior to receiving the results for their COVID-19 tests. Because of this, women receiving a negative COVID-19 result after enrollment and were subsequently deemed ineligible to participate in the study and were not included in the total number of participants. Milk and swabs of the nipple/areola (“breast skin swabs”) were collected as previously described (3). COVID-19 signs and symptoms (e.g., cough, fever, congestion, fatigue, malaise, difficulty breathing, chest pain, loss of smell and/or taste, and diarrhea) were recorded at study enrollment and at each sample collection. Milk samples collected prior to December 2019 from 5 healthy women located in the greater Rochester, NY area for general assay development were used as prepandemic control samples.

### Laboratory Analysis

Total RNA was extracted from the first three milk samples collected from 47 women not previously reported on and extraction controls using the Quick-DNA/RNA Viral MagBead kit (Zymo Research, Irving, CA). Briefly, 200 µL of whole milk were mixed with 200 µL of 2X DNA/RNA Shield (Zymo Research) and incubated for 10 min prior to extraction following manufacturer’s instructions. Total RNA was then used as the input for the CDC-designed SARS-CoV-2 reverse transcription quantitative polymerase chain reaction (RT-qPCR) assay targeting two regions of the SARS-CoV-2 nucleocapsid gene, validated for use with human milk and replicated across two laboratories (University of Idaho and University of Rochester) as previously described (3). Per the CDC protocol, samples with Ct values <40 were considered positive. It is noteworthy that we did not reanalyze samples reported previously (3) for SARS-CoV-2 RNA because assay parameters had not changed. Total RNA was also extracted from swabs collected prior to the first three milk collections of 35 women not previously reported on and analyzed using the same RT-qPCR assay used for human milk with analysis only occurring at the University of Idaho. For breast swabs, swab heads were immersed in 400 µL 1X DNA/RNA Shield (Zymo Research), pulse vortexed for 20 seconds, incubated for 10 min, centrifuged at 500 x g for 1 min at 22°C, and then up to 400 μL of the liquid were used as input for RNA isolation. For extraction negative controls, 400 μL 1X DNA/RNA Shield were used as the input.

Concentrations of milk-borne anti-RBD IgA were determined in duplicate from delipidated milk using an enzyme-linked immunoassay (ELISA) as previously described (17) with the following modifications. Microtiter plates (Nunc Maxisorb, ThermoFisher Scientific) were coated with SARS-CoV-2 spike glycoprotein RBD (Sino Biological, Beijing, China) and blocked with 1% human serum albumin (HSA) (Millipore, Burlington, MA) in phosphate buffered saline containing 0.05% Tween-20 (PBS-T). Serum with known high anti-RBD IgA concentration (Ray Biotech, Peachtree Corners, GA) was used as a standard with dilution series ranging from 1:100 to 1:1,000,000 and milk samples were diluted 1:2 with 1% HSA and incubated in coated wells overnight at 4°C. After washing with PBS-T, bound antigen-specific antibodies were detected by incubating wells with horseradish peroxidase-conjugated polyclonal goat anti-human IgA antibody (Bethyl Laboratories, TX, USA), followed by washing with PBS-T and developing color with BD OptEIA reagent kit (Becton Dickinson). The color reaction was stopped after 10 min by adding 0.18 N sulfuric acid, and absorbance was read at 450 nm using a 96-well plate reader (BioTek, VT, USA). A standard curve was generated by fitting a 5PL equation to standard dilution series absorbances using plate reader software (Take5, BioTek, VT, USA), and sample concentrations were back calculated. Specific antibody concentrations are expressed based on standard serum (1 AU corresponds to the amount of specific IgA in 1:10,000 dilution of the standard serum). A positive cutoff threshold for positivity/antigen-specific binding was set as the sum of the mean and 2 times the standard deviation of RBD-specific IgA in prepandemic milk samples. SARS-CoV-2 RBD-specific IgA concentration for some of the samples were included in a previous study but were measured again in the present study for consistency. One participant’s milk sample was not tested for IgA due to insufficient volume.

### Statistical Analysis

R version 3.6.1 (18) and GraphPad Prism 9 were used for data analyses. The exact binomial test was used to calculate confidence intervals. The R package lmer (19) was used to perform univariate logistic regression to assess the relationships between the detection of viral RNA on breast skin swabs and the incidence of maternal respiratory symptoms or household COVID-19 symptoms/diagnosis with individual included as a random effect. Wilcoxon signed-rank test was used to assess the difference in anti-RBD IgA concentration between the first and second week of illness. Statistical significance was declared at *P*<.05.

## Results

### Cohort Characteristics

Samples were collected from 64 women diagnosed with COVID-19 (**Supplementary Figures 1, 2**). Participant characteristics are presented in **Table 1**. Briefly, median age was 33 years (interquartile range [IQR], 30-36), and median time postpartum was 18 weeks (IQR, 2-32). Symptomatic and asymptomatic COVID-19 was reported in 83 (*n*=53) and 17% (*n*=11) of participants, respectively. Overall, relative to the day of diagnostic testing, we collected 78 milk samples from 40 women within the first week; 120 samples from 58 women between days 8 and 21; 89 samples from 47 women between days 22 and 56; and 29 samples from 29 women between days 57 and 106. In total, 316 milk samples were collected from 64 women. It is noteworthy that we have previously reported selected results from 37 milk samples produced by 17 of these women (3).

**Table 1:** Selected characteristics and behaviors of study participants.

Characteristic	No. (% or IQR)
Participants	64
Age,^a^ median, y	33 (30-36)
Race	
Asian	1 (2)
Black	3 (5)
White	49 (77)
Other	8 (12)
Not reported	3 (5)
BMI,^b^ median, kg/m^2^	27 (23-31)
Parity,^a^ median	2 (1-3)
Time postpartum,^a^ median, wk	18 (2-32)
Breastfeeding status^c^	
Exclusively breastfeeding	19 (37)
Mixed feeding	33 (63)
COVID-19 symptoms	
Symptomatic	53 (83)
Asymptomatic	11 (17)
Infants tested for COVID-19^d^	20 (38)
Positive diagnosis	7 (35)

IQR, interquartile range; BMI, body mass index; COVID-19, coronavirus disease 2019. ^a^, Missing data from 1 participant; ^b^, Missing data from 4 participants; ^c^, Missing data from 12 participants; ^d^, Missing data from 11 participants; Percentages may not sum to 100 due to rounding.

### Evaluation of Milk and Breast Skin Swabs for the Presence of SARS-CoV-2 RNA

Here, we analyzed the first three milk samples (*n*=141) collected from 47 women and the first three breast skin swabs (*n*=99) collected prior to milk collection from 35 women not described in our previous report (3) for SARS-CoV-2 RNA (range, 0-37 days from diagnostic test). As with our prior report, all milk samples tested negative for SARS-CoV-2 in both laboratories. In contrast, while 71% (70/99; 95% CI, 61-79%) of the swabs collected before breast washing tested negative for SARS-CoV-2, 29% (29/99) generated Ct values that varied in degree of positivity (**Supplementary Table 1**). However, 27/29 (95% CI, 77-99%) of the companion swabs collected after breast washing tested negative for SARS-CoV-2. The two swabs that retained some degree of positivity after washing had a 70-80% reduction in the estimated viral load.

Using these swab data combined with our previously published swab data (3), we evaluated whether maternal respiratory symptoms of COVID-19 (cough, dyspnea, rhinorrhea/nasal congestion, and sneezing) or presence of household COVID-19 were related to the detection of SARS-CoV-2 RNA on the breast skin. Among the four maternal respiratory symptoms examined, only cough was related to the detection of viral RNA (odds ratio, OR, 4.78; 95% CI, 1.59-14.38; *P*<.01; 51% of swabs with cough versus 19% without cough) (**Table 2**). The presence of at least one other household member with COVID-19 was also associated with increased likelihood of detection of viral RNA on breast skin swabs (OR, 6.67; 95% CI, 1.79-24.92; *P*<.01; 53% of swabs with household COVID-19 versus 18% without household COVID-19).

**Table 2:** Association of respiratory signs/symptoms and viral RNA presence on the breast skin.

Sign/symptom	OR (95% CI)
Cough	4.78 (1.59-14.38)**
Dyspnea	0.91 (0.15-5.52)
Rhinorrhea/nasal congestion	2.94 (0.86-10.07)
Sneeze	0.22 (0.01-3.26)

**P<.01; n=116 breast skin swabs from 43 participants; OR, odds ratio; CI, confidence interval.

### Longitudinal Assessment of Milk Specific IgA

**Figure 1:**
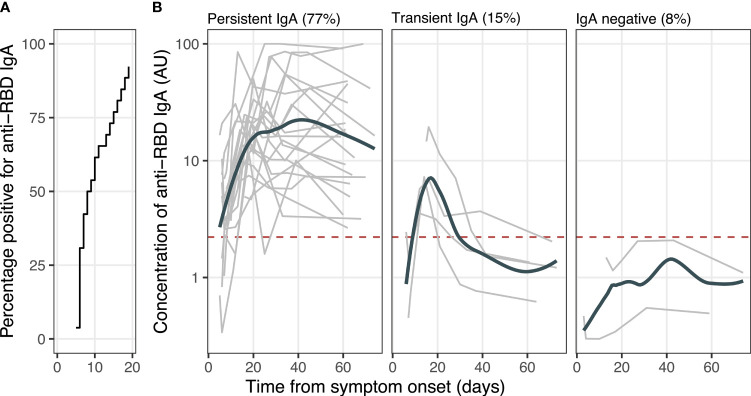
Temporal dynamics of milk anti-RBD IgA. **(A)** Proportion of women with milk anti-RBD IgA. **(B)** Concentration of milk anti-RBD IgA. The gray lines represent individuals (*n*=26); bolded lines represent the group LOESS curves; and horizontal dashed red line denotes the cutoff for assay positivity/limit of antigen-specific binding.

Assays for detecting IgA specific to the S glycoprotein RBD were conducted on 316 milk samples collected from all 64 women; 75% (95% CI, 70-79%) of these samples contained detectable anti-RBD IgA. The maximum concentration of anti-RBD IgA was two-fold higher in symptomatic women in comparison to asymptomatic women, although this difference was not statistically significant (*P*=.0610; symptomatic - 22.8 ± 27.1 AU, average ± standard deviation; asymptomatic - 11.2 ± 15.7 AU). Longitudinal analysis of samples collected from women who provided repeated samples for at least 2 months following onset of symptoms (n=24) or positive test (for asymptomatic women, n=2) demonstrated an increase (*P*=.02) in the concentration of anti-RBD IgA from the first to second week following onset of symptoms/positive test; 92% (24/26) of these women produced milk containing anti-RBD IgA by day 19 (**Figure 1A**). Of these 26 women, 77% (n=20) produced milk with anti-RBD IgA for 2 months or longer (“persistent IgA”), whereas 15% (n=4) produced milk without persistent levels of detectable anti-RBD IgA (“transient IgA”; **Figure 1B**). Of the two asymptomatic women with longitudinal data, one displayed persistent IgA while the other was IgA negative.

## Discussion

Consistent with the preponderance of available evidence, we found no indication of SARS-CoV-2 in milk produced by women with COVID-19. Some breast skin swabs were found to contain detectable SARS-CoV-2 RNA, almost all of which were collected prior to washing the breast. Detection of SARS-CoV-2 RNA on the unwashed breast was related to the presence of maternal cough and others in the household with COVID-19.

Importantly, we detected a rapid, robust, and durable anti-RBD specific IgA response in the majority of mothers’ milk. Our longitudinal study substantially extends prior knowledge by demonstrating: *(I)* the vast majority (92%) of women with COVID-19 have anti-RBD IgA in their milk; *(II)* concentrations of anti-RBD IgA increase during the first weeks following onset of symptoms or, if asymptomatic, following the day of diagnostic test; and *(III)* anti-RBD IgA is present in milk produced by most infected women for at least 2 months.

Our finding of a persistent antibody response in milk produced by women with COVID-19 is reassuring as it suggests passive immunity is likely conferred to recipient infants for at least 2 months after maternal infection. Passive immunity *via* milk is particularly important for breastfeeding children, including infants and neonates, as COVID-19 vaccines have yet to be approved for these populations. Further, our results showing a sustained anti-RBD antibody response in milk may have implications for the durability of vaccine-induced milk antibodies. Indeed, similar to SARS-CoV-2 infection and maternal vaccination against other respiratory pathogens (20, 21), recent data demonstrate that in the days and weeks following maternal COVID-19 vaccination, a robust IgG-dominant milk antibody response is induced (22–25), although the longer-term durability of the milk-borne antibody response remains to be elucidated. Similarly, the mechanisms underlying the persistence or lack of specific antibodies in human milk are not well understood, but may be related to differences in the course of the infection, recurrent exposures and/or the migration of long-lived plasma cells from mucosal sites to the mammary gland (26–28)

Our previous (3) and current findings on the detection of SARS-CoV-2 RNA on the breast skin of a small number of participants prior to cleaning may provide an explanation as to why some milk samples in prior studies have yielded positive results for SARS-CoV-2 RNA *via* RT-qPCR. It is worth noting that only low titers of viral RNA were detected on the positive breast skin swab samples (**Supplementary Table 1**). Washing of the breast appeared effective in removing the RNA in almost all cases examined. Unfortunately, since the breast skin swabs utilized for viral RNA detection were inactivated prior to RT-qPCR, we were unable to examine whether the RNA represented viable virus or remnants of viral RNA and poses a potential risk of maternal-to-child transmission. However, it is notable that numerous studies examining neonatal outcomes during maternal SARS-CoV-2 infection have not found evidence of SARS-CoV-2 transmission *via* breastfeeding (12, 13, 29–33).

We recognize that this study was limited by self-reported COVID-19 diagnostic test results for most women, self-collection of samples, and lack of individuals with severe COVID-19 that required hospitalization, and an inability to assess functional immunity or virus neutralization. However, strengths of the study include a relatively large sample size, longitudinal sampling, collection methodology employing best practices for human milk research (34), and use of assays validated for human milk (3) to detect SARS-CoV-2 RNA and profile the dynamics of milk-specific IgA.

In summary, we found no evidence of SARS-CoV-2 in milk and documented the presence of anti-RBD IgA that persisted for at least 2 months in milk produced by most study participants. Beyond the health impacts of human milk as a source of nutrition, these data suggest that, on balance, human milk is not a source of SARS-CoV-2 transmission and may provide lasting passive immunity. Our findings also provide additional support for recommendations that lactating women with COVID-19 continue to breastfeed while they and others in the household take precautions, such as hand and respiratory hygiene, to prevent transmission *via* respiratory droplets (35).

## Data Availability Statement

The raw data supporting the conclusions of this article will be made available by the authors, without undue reservation.

## Ethics Statement

This multi-institutional study was reviewed and approved by the institutional review boards of the University of Idaho (20-056, 20-060), University of Rochester Medical Center (1507), University of California, San Francisco (20-30410), Brigham and Women’s Hospital (2020P000804), University of Arkansas for Medical Sciences (260939), and Tulane University (2020-602). All participants gave written informed consent.

## Author Contributions

Concept and design: RP, JW, KJ, CM, MMa, SL, CB-L, LY, AA, MB, SG, VF, MMc, MKM, and AS. Acquisition, analysis, or interpretation of data: All authors. Drafting of the manuscript: RP and JW. Critical revision of the manuscript for important intellectual content: All authors. Statistical analysis: RP, JW, and AS. Obtained funding: RP, JW, KJ, CM, MMa, SL, CB-L, LY, AA, MB, SG, VF, MMc, MKM, and AS. Administrative, technical, or material support: RP, JW, KJ, SG, VF, MMc, MKM, and AS. Supervision: RP, JW, KJ, CM, MMc, MKM, and AS. All authors contributed to the article and approved the submitted version.

## Funding

This work was supported, in part, by the Bill and Melinda Gates Foundation (INV-016943, INV-017035). Under the grant conditions of the Foundation, a Creative Commons Attribution 4.0 Generic License has already been assigned to the Author Accepted Manuscript version that might arise from this submission. Additional support for this work was provided by the National Science Foundation (IOS-BIO 2031753, 2031715, 2031888, 2031761), US National Institutes of Health (R01 HD092297-03, U01 AI131344-04S1), Washington State University Health Equity Research Center, USDA National Institute of Food and Agriculture (Hatch project IDA01643), and the Arkansas Children’s Research Institute. In-kind support was provided by Medela and Milk Stork. The funding agencies had no role in the collection, management, analysis, and interpretation of the data; preparation, review, or approval of the manuscript; and decision to submit the manuscript for publication.

## Conflict of Interest

The authors declare that the research was conducted in the absence of any commercial or financial relationships that could be construed as a potential conflict of interest.

## Publisher’s Note

All claims expressed in this article are solely those of the authors and do not necessarily represent those of their affiliated organizations, or those of the publisher, the editors and the reviewers. Any product that may be evaluated in this article, or claim that may be made by its manufacturer, is not guaranteed or endorsed by the publisher.
