# Supplementary material for: Milk From Women Diagnosed With COVID-19 Does Not Contain SARS-CoV-2 RNA but Has Persistent Levels of SARS-CoV-2-Specific IgA Antibodies
Source: Front Immunol. 2021 Dec 23;12:801797. doi: 10.3389/fimmu.2021.801797 (PMC8733294; doi:10.3389/fimmu.2021.801797)
Supplement: Supplementary file 1 [file DataSheet_1.docx]

Supplementary Material


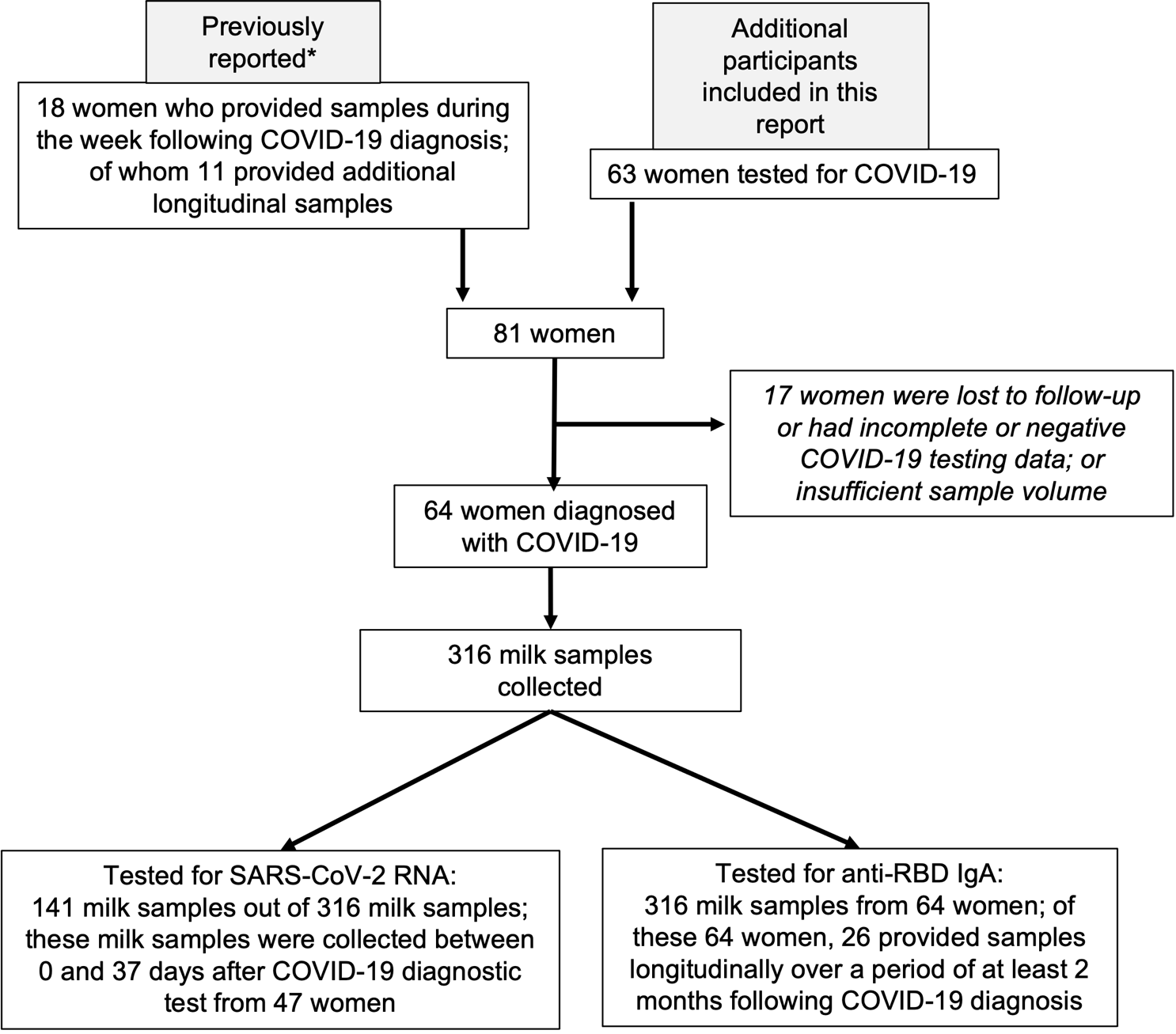


**Supplementary Figure 1.** Flowchart of number of participants and milk samples per analysis. *Pace RM, Williams JE, Järvinen KM, et al. Characterization of SARS-CoV-2 RNA, antibodies, and neutralizing capacity in milk produced by women with COVID-19. mBio. 2021;12(1):e03192–20. doi:10.1128/mBio.03192-20.


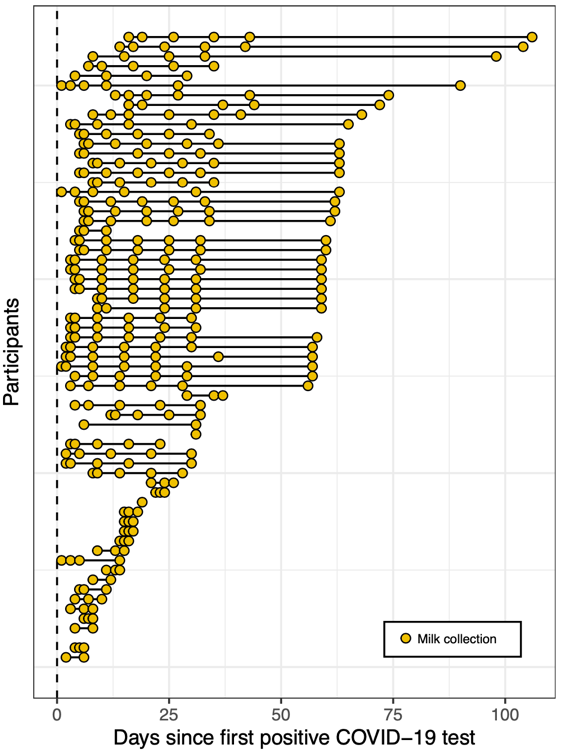


**Supplementary Figure 2.** Overview of timing of milk samples collected by the 64 breastfeeding women participating in this study.

**eTable 1.** **Breast skin swabs with evidence of SARS-CoV-2 RNA**.

| Participant | Collection | Before Breast Washing | | | After Breast Washing | | |
| --- | --- | --- | --- | --- | --- | --- | --- |
|  |  | **Result** | **N1 Ct** | **N2 Ct** | **Result** | **N1 Ct** | **N2 Ct** |
| LC53 | First  Second  Third | Indeterminate  Negative  Negative | 36.3/nd  nd  nd | 37.9/nd  nd  nd | Negative  -  - | nd  -  - | nd  -  - |
| LC21 | First  Second  Third | Negative  Negative  Indeterminate | nd  nd  36.9/nd | nd  nd  35.9/nd | -  -  Negative | -  -  nd | -  -  nd |
| LC20 | First  Second  Third | Indeterminate  Negative  Indeterminate | nd  nd  nd | 36.7/nd  nd  36.9/nd | Negative  -  Negative | nd  -  nd | nd  -  nd |
| LC46 | First  Second  Third | Positive  Positive  Indeterminate | 29.3/29.  33.7/34.1  nd | 29.5/30.5  33.6/34.3  36.5/nd | Negative  Negative  Negative | nd  nd  nd | nd  nd  nd |
| LC40 | First  Second  Third | Negative  Positive  Positive | nd  34.4/35.1  35.1/36.4 | nd  35.2/37.3  34.2/35.7 | -  Negative  Negative | -  nd  nd | -  nd  nd |
| LC41 | First  Second  Third | Indeterminate  Negative  Negative | 35.4/nd  nd  nd | 36.7/nd  nd  nd | Negative  -  - | nd  -  - | nd  -  - |
| LC51 | First  Second  Third | Negative  Positive  Negative | nd  32.3/35.0  nd | nd  33.2/33.7  nd | -  Negative  - | -  nd  - | -  nd  - |
| LC48 | First  Second^a^  Third | Negative  Indeterminate  Negative | nd  nd  nd | nd  36.7/37.4  nd | -  Negative  - | -  nd  - | -  nd  - |
| LC32 | First  Second  Third | Positive  Indeterminate  Negative | 36.6/37.0  nd  nd | 36.7/35.7  36.7/nd  nd | Negative  Negative  - | nd  nd  - | nd  nd  - |
| LC56 | First  Second  Third | Negative  Indeterminate  Negative | nd  nd  nd | nd  37.2/nd  nd | -  Negative  - | -  nd  - | -  nd  - |
| LC39 | First  Second  Third | Negative  Negative  Positive | nd  nd  33.0/32.5 | nd  nd  33.8/34.5 | -  -  Negative | -  -  nd | -  -  nd |
| LC33 | First  Second  Third | Indeterminate  Indeterminate  Negative | 36.1/nd  36.2/nd  nd | 32.7/nd  37.8/nd  nd | Negative  Negative  - | nd  nd  - | nd  nd  - |
| LC43 | First  Second  Third | Indeterminate  Negative  Negative | 36.3/nd  nd  nd | 37.7/nd  nd  nd | Negative  -  - | nd  **-**  **-** | nd  **-**  **-** |
| LC35 | First  Second  Third | Indeterminate  Positive  Negative | 37.3/nd  36.1/36.2  nd | nd  36.3/37.0  nd | Negative  Negative  - | nd  nd  **-** | nd  nd  **-** |
| LC23 | First  Second  Third | Positive  Positive  Positive | 29.6/29.6  30.4/30.8  34.7/35.1 | 29.6/29.8  30.1/30.3  34.7/36.2 | Positive  Negative  Negative | 31.9/32.0  nd  nd | 33.0/33.1  nd  nd |
| LC55 | First  Second  Third^a^ | Indeterminate  Negative  Positive | 34.2/nd  nd  34.6/36.4 | 34.9/35.1  nd  34.3/34.6 | Indeterminate  -  Negative | 36.0/nd  -  nd | 37.0/37.2  -  nd |
| LC11 | First  Second  Third | Indeterminate  Indeterminate  Indeterminate | 36.1/nd  36.2/nd  35.1/nd | 36.8/nd  35.4/nd  35.9/nd | Negative  Negative  Negative | nd  nd  nd | nd  nd  nd |

**Table footnotes:** Ct values for RT-qPCR duplicates of each SARS-CoV-2 target are given. nd, not detected; -, not assayed; ^a^, post-washing swab was collected after milk collection, rather than before.
